# A Multicenter, Randomized, Double-Blind, Split-Body Clinical Trial Evaluating the Efficacy and Outcomes of a Topical Product Pre and Post Aesthetic Surgical Body Procedures

**DOI:** 10.1093/asjof/ojac054

**Published:** 2022-06-10

**Authors:** Laurie A Casas, R Brannon Claytor, Kamakshi R Zeidler, Sachin M Shridharani, Steven R Cohen, Julie J Khanna, Daniel J Gould, Essie K Yates, Shantel Lultschik, Michaela Bell, Alan D Widgerow

**Affiliations:** Section of Plastic & Reconstructive Surgery, The University of Chicago Medicine, Chicago, IL, USA; Division of Plastic Surgery, Main Line Health Systems, Bryn Mawr, PA, USA; Division of Plastic Surgery, Washington University School of Medicine, St. Louis, MO, USA; Division of Plastic Surgery, University of California, San Diego, CA, USA; NOVA Southeastern University, Fort Lauderdale, FL, USA; Alastin Skincare, Inc., Carlsbad, CA, USA

## Abstract

**Background:**

Skin preconditioning prior to and following procedures, has previously been demonstrated to hasten and optimize healing, and decrease the symptoms and signs associated with invasive surgery. These trials involved the use of multiple topical products. In an effort to control costs and to increase patient compliance, a single surgical product was developed, with actives aimed at decreasing swelling, bruising, induration, and internal fibrous banding.

**Objectives:**

This multi-center trial was designed to measure the efficacy of this single product in these mentioned parameters.

**Methods:**

A double-blind, randomized, split body, clinical study was undertaken in 29 patients involving 38 surgical procedures. Assessments included photography, biopsies, ultrasound imaging, and blinded investigator and participant assessments.

**Results:**

Differentiated results between test comparator sides became apparent at postop day 10-14 (as previously observed). Thus, blinded investigator and participant assessment scores demonstrated statistical significance exclusive to the test product side at postop day 10-14 for ecchymoses and then extending to skin discoloration, edema, induration and subcutaneous fibrous banding, at weeks 3, 4, 6, and 12. Ultrasound evaluation confirmed the earlier dissolution of fibrous banding on the test side in the subcutaneous tissue at the 3-6-week postop period. In addition, biopsies assessing the pre-conditioned period prior to surgery confirmed that the topical test product stimulated a remodeled extracellular matrix without comparative changes on the opposite side.

**Conclusions:**

A single peri-surgical product designed for the use with invasive surgery produced significant differences in ecchymosis, skin discoloration, edema, induration and ongoing resolution of fibrous banding over many weeks. This study validation provides an additional adjunct to surgical procedures.

**Level of Evidence: 2:**

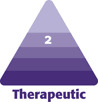

Postsurgical recovery time and optimized healing are paramount to both physicians and patients alike. A recent series of clinical studies have been completed and published with respect to topical products being used pre and post aesthetic surgical procedures to lessen adverse responses such as edema, ecchymosis, skin discoloration, induration, subcutaneous fibrous banding, and pain/discomfort.^1-5^

Other clinical studies have shown that wound bed preparation using an anhydrous topical gel, Regenerating Skin Nectar with TriHex Technology (RSN; Alastin Skincare, Inc., Carlsbad, CA), accelerates healing from laser injury and provides evidence of extracellular matrix remodeling when used pre and post aesthetic surgical procedures.^6,7^ Many of these indications necessitated the use of separate topical applications with a unique set of actives that were individually validated. The synergistic combination of these actives with the addition of a component for scar alleviation has been formulated for use before and after surgery with the intention of improving patient compliance and patient-reported recovery outcome measures (PROMs) following elective cosmetic surgical procedures. Table 1 lists the actives and clinical effect. This study evaluated one topical product compared with a bland moisturizer, pre and post surgical procedures, for improvement in recovery outcomes.

**Table 1.** R&R Actives and Clinical Effect

Presurgical and postsurgical: topical ingredients	Clinical effect
• Tripeptide-1 Hexapeptide-12 (TriHex) • Phosphatidylserine• Oleuropein	A blend of active peptides and key ingredients that works with the skin to support clearing out damaged elastin and collagen
• Hexapeptide-11 • Phosphatidylserine	Fat dissolution (autophagy)
• Tripeptide-1 • Hexapeptide-12 • Hexapeptide-11 • Lactoferrin • Phosphatidylserine	Bruising
• Lactoferrin • Xylitol • Oleuropein	Anti-microbial
• Phytoene, phytofluene • Oleuropein • Hydroxymethoxy-phenyl decanone • Naringenin	Swelling
• Tetrandrine • Centella asiatica • Oleuropein • Phytoene, phytofluene inflammation • Naringenin	Scarring

R&R, ReFORM & RePAIR with TriHex Technology (Alastin Skincare, Inc., Carlsbad, CA).

## METHODS

This double-blind, randomized, split-body, clinical study was approved by a private institutional review board, Advarra, Inc. (Columbia, MD). The study occurred over 11 months from April 2021 to February 2022, and the enrollment period occurred over 6 months between April 2021 and September 2021. There were 7 clinical sites within the United States and 1 in Canada. Each clinical site had the same board-certified surgeon performing the procedures. Inclusion criteria included men and women, 25 to 70 years of age, electing an aesthetic surgical body procedure and willing to apply 2 different topicals to the surgical area before and after the procedure and refrain from any other aesthetic procedures or topical products, outside the investigator’s standard of care, during the duration of the study. Potential participants were excluded from the study who had an uncontrolled, clinically significant medical disorder or poor health, which, in the opinion of the investigator, may have interfered with the procedure or accurate evaluation of the skin characteristics or inhibited wound healing; unwilling or unable to comply with the requirements of the protocol and use of topicals with active ingredients in the surgical/procedural area, which, in the opinion of the investigator, may interfere with the outcomes; or a previous hypersensitivity to any of the ingredients in the study products. Pregnant or lactating participants were also excluded as well as participants planning on becoming pregnant during the study duration.

In total, 29 participants underwent 38 bilateral procedures. All enrolled participants were provided 2 randomized identical blinded bottles, to apply one to the right and the other to the left side of the surgical area. Randomization of products was completed through excel and products were placed in a sealed bag with a kit number. Kits were distributed by a clinical study team member, in chronological order. Study products consisted of ReFORM & RePAIR with TriHex Technology (R&R; Alastin Skincare, Inc., Carlsbad, CA) and Bland Moisturizer: Cetaphil Lotion (Galderma, Fort Worth, TX). Depending on the procedural area, each participant was instructed to apply 2 to 3 pumps of the designated product to the side of the body, twice a day for at least 2 weeks before the surgical procedure and for 12 weeks postoperatively. In order to not cross-contaminate the right and left sides, participants were instructed to either use separate hands to apply the designated topical or wash hands in between the application of sides. There was not an instructed motion or recommended pressure to the application on either side. Postoperatively, participants were instructed to use the designated topical in the same surgical areas and to include the skin at the incision. Garments and dressings were specified according to the surgeons’ postop care. Additional adjuncts, manual lymphatic drainage (MLD), were performed on both sides according to the surgeons’ postop instruction. Participants were evaluated at 7 postoperative intervals: days 1-3, 5-7, 10-14, 21-25, 28-30, 42-50, and at 12 weeks. At specified timepoints, the following procedures were completed.

### Biopsies

Five participants consented to a 3-mm punch biopsy on the right and left sides of surgical area before applying the randomized topicals and on the day of the surgical procedure. Biopsies were performed by the operating surgeon, in the area of the skin to be excised during surgery. This was used as an assessment of skin preconditioning before surgery.

### Blinded Investigator or Designee Assessment

At every postoperative visit, the investigator or designee completed an assessment of the right and left sides using a 5-point scale (0—none; 1—barely perceptible, visually, or palpably; 2—mild; 3—moderate; and 4—severe) for the following symptoms: ecchymosis, swelling, skin discoloration, induration, and subcutaneous fibrous banding. The visual analog scale (VAS) 0-10 pain scale was administered in person to the participant at each visit.

### Blinded Participant Assessment and Questionnaire

At every postoperative visit, the participant completed the following assessment of the right and left sides using a 5-point scale (0—none; 1—barely perceptible, visually, or palpably; 2—mild; 3—moderate; and 4—severe) for the following symptoms: ecchymosis, swelling, skin discoloration, induration, and subcutaneous fibrous banding. A blinded study team member would assist each participant with the clinical definition/indication of each symptom. Additionally, participants completed a questionnaire designating either right side, left side, or no difference for the following: less swelling, less numbness, less bruising/discoloration, less pain/discomfort, feels softer and more flexible, and better skin texture.

### Ultrasound Imaging

The Episcan I-200 with a 35 MHz probe (Longport, Inc., USA, Chadds Ford, PA) was used at 2 clinical sites for ultrasound imaging up to 5 mm in depth. Ultrasound images were captured by the same board-certified surgeon or physician assistant at all visits up to postoperative days 42-50. Each ultrasound was taken in the same measured location within the surgical area, remote from the biopsy sites, if applicable. Measured locations were selected by the surgeon at baseline, based on reproducibility and area of most disruption.

Locations per procedure are as follows:

- Breast: at the intersection of the anterior axillary line and the inframammary crease;
- Abdominoplasty: at the intersection of patients’ waistline and lateral border of the *rectus abdominis* muscle;
- Axillary liposuction: at the intersection of posterior axillary line and the inframammary fold (IMF);
- Hip/flank liposuction with patient prone: at the intersection of patients’ waistline and the vertical line drawn for the posterior axillary line;
- Lateral thigh liposuction: 2 measured locations on each lateral thigh; between 20 and 32 cm above the lateral knee, measured 7-9 cm from the midline of the patella.

### Photography

At every visit, photography of the participant was completed. Each investigator took their own standardized photography, according to their practice. One clinical site used a 3D camera system, LifeViz Body & Breast (Quantificare, Cummings, GA).

### Breaking the Blind

At every postprocedure visit, each participant was given the option to proceed with the blinded study product application, opt out, or break the randomization and use the topical side of preference based on the recovery as per IRB instructions. This study was conducted in accordance with the World Medical Association Declaration of Helsinki statement of ethical principles for medical research involving human subjects, including research on identifiable human material and data. Full written informed consent was acquired from each participant, for use and analysis of their data in the study.

## RESULTS

Twenty-nine females, age range 31 to 69 years, mean age 48 years, Fitzpatrick skin types (type I [n = 2], type II [n = 7], type III [n = 7], type IV [n = 7], type V [n = 5], and type VI [n = 1]), completed the study. Participants were followed through 12 weeks postop and completed 7 postoperative visits: days 1-3, 5-7, 10-14, 21-25, 28-30, 42-50, and 12 weeks. There were 38 surgical procedures evaluated in this study. Participants having 2 bilateral procedures during the surgery were evaluated as 2 separate procedural areas. Table 2 lists each surgical procedure.

**Table 2.** Surgical Procedures Evaluated in the Study

Number	Surgical procedure
3	Abdominoplasty
4	Abdominoplasty with PAL of the abdomen, hips, and flanks
1	Abdominoplasty with PAL of the abdomen, hips, and flanks; bilateral explant with fat transfer to bilateral breasts
6	Abdominoplasty with mastopexy
2	Bilateral breast reduction
1	Bilateral mastopexy
2	Bilateral breast augmentation with mastopexy
1	Capsulectomy with breast implant exchange
1	Bilateral breast augmentation
1	Bilateral secondary breast augmentation
4	PAL to anterior thighs
1	Vaser (ultrasound) liposuction to thighs
1	Laser liposuction to the neck
3	Liposuction mechanical to the axilla
3	Vaser (ultrasound) liposuction to hips and flanks
1	J-Plasma to submental
1	PAL to bilateral calves, ankles, and medial knees, liposuction with J-plasma to bilateral anterior and inner thighs
1	Liposuction with J-plasma to the bilateral upper back and flanks; J-plasma to the total abdomen; autologous fat transfer to bilateral breast
1	Bilateral limited incision brachioplasty (axillary)

PAL, power-assisted liposuction.

### Assessment Statistical Analyses

For each procedure, at each time point, the score difference between the side treated with bland moisturizer and the side treated with R&R application was computed. To test for a significant difference in the outcome between the 2 sides, both parametric test (ie, paired *t* test) and nonparametric test (ie, signed rank test) were performed to ensure that the data analysis was robust regardless of the statistical methods performed.

### Blinded Investigator/Designee Assessments

**Figure 1.**
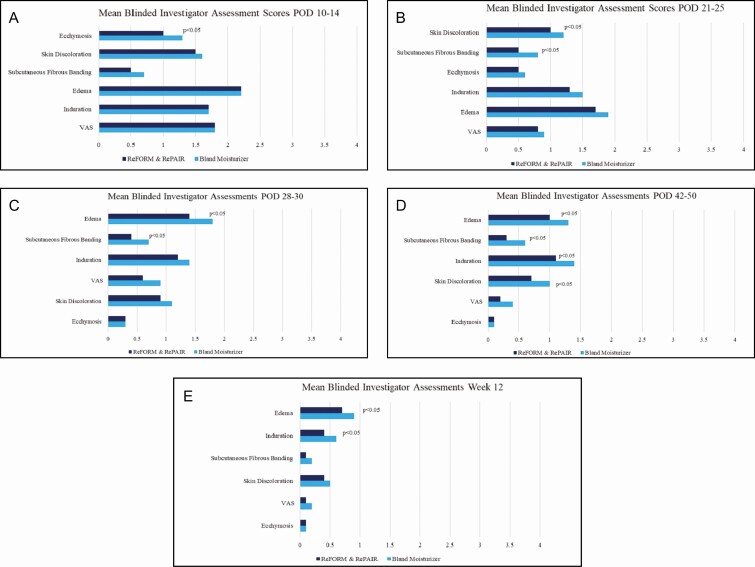
Mean blinded investigator assessment scores: (A) POD 10-14, (B) POD 21-25, (C) POD 28-30, (D) POD 42-50, and (E) week 12. POD, postoperative days; VAS, visual analog scale.

At each follow-up visit, the blinded investigator assessed the right and left sides of the procedural area. At the following postop days (POD), there was a significant statistical improvement in the side using R&R over the bland moisturizer. It is interesting to note that at no time point measured did the comparator show statistical advantage over R&R from the blinded investigator perspective (Figure 1):

- Ecchymosis at POD 10-14 (difference = 0.2, standard deviation [SD] = 0.6, *P*-value = 0.0296)
- Skin discoloration at POD 21-25 (difference = 0.2, SD = 0.5, *P*-value = 0.0141)
- Subcutaneous fibrous banding at POD 21-25 (difference = 0.3, SD = 0.5, *P*-value = 0.0025)
- Subcutaneous fibrous banding at POD 28-30 (difference = 0.3, SD = 0.6, *P*-value = 0.0057)
- Edema at POD 28-30 (difference = 0.4, SD = 0.7, *P*-value = 0.0035)
- Skin discoloration at POD 42-50 (difference = 0.3, SD = 0.7, *P*-value = 0.0096)
- Edema at POD 42-50 (difference = 0.3, SD = 0.7, *P*-value = 0.0056)
- Subcutaneous fibrous banding at POD 42-50 (difference = 0.4, SD = 0.9, *P*-value = 0.00213)
- Induration at POD 42-50 (difference = 0.2, SD = 0.6, *P*-value = 0.0222)
- Edema at week 12 (difference = 0.2, SD = 0.6, *P*-value = 0.0412)
- Induration at week 12 (difference = 0.2, SD = 0.6, *P*-value = 0.0460)

### Blinded Participant Assessments

**Figure 2.**
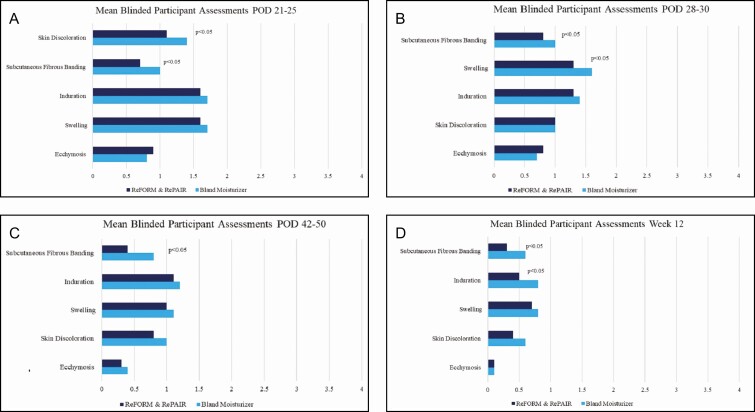
Mean blinded participant assessment scores: (A) POD 21-25, (B) POD 28-30, (C) POD 42-50, and (D) week 12. POD, postoperative days.

Participants assessed each side of the procedural area at every follow-up visit. The side using R&R had a significant statistical improvement over bland moisturizer at the following POD for skin discoloration, subcutaneous fibrous banding, swelling, and induration (Figure 2):

- Skin discoloration at POD 21-25 (difference = 0.3, SD = 0.6, *P*-value = 0.0161)
- Subcutaneous fibrous banding at POD 21-25 (differences = 0.3, SD = 0.6, *P*-value = 0.0121)
- Swelling POD 28-30 (differences = 0.3, SD = 0.9, *P*-value = 0.0409)
- Subcutaneous fibrous banding at POD 28-30 (differences = 0.3, SD = 0.7, *P*-value = 0.0512)
- Subcutaneous fibrous banding at POD 42-50 (differences = 0.3, SD = 0.8, *P*-value = 0.0240)
- Subcutaneous fibrous banding at week 12 (differences = 0.3, SD = 0.7, *P*-value = 0.0328)
- Induration at week 12 (difference = 0.3, SD = 0.4, *P*-value = 0.0031)

At POD 1-5, participants graded swelling on the bland moisturizer side lower which had a statistical improvement over R&R. This was the only time point and postop symptom that the bland moisturizer was statistically significant over R&R.

### Blinded Participant Questionnaire and Statistical Analyses

Missing or non-applicable responses were excluded from the analysis, and the percentage of each response category was calculated at each POD. Chi-square tests were performed to detect any significant differences in the responses between R&R and bland moisturizer. A significantly greater percentage of participants reported less bruising and skin discoloration (*P* < 0.0001), less pain/discomfort (*P* = 0.0084), and feels softer and more flexible (*P* = 0.0387) on the R&R side over the bland moisturizer side.

### Ultrasound Imaging

Two clinical sites completed ultrasound images at every visit excluding week 12. All ultrasound images were analyzed by a blinded independent consultant from Longport, Inc. The following were analyzed:

- Dermal thickness—the dermis is expected to increase in thickness following all procedures. The degree of thickness increases as well as the speed of recovery is determined by the impact of the procedure with the least increase and fastest recovery to normal being preferred.
- Dermal density—ultrasound does not directly measure density, but the intensity of the reflected signal or brightness is related to the density if no other variable is introduced. The brightness of the dermis is expected to reduce following all procedures. The degree of brightness change is determined by the impact of the procedure with the least decrease and fastest recovery to normal being preferred. Three measures of brightness/density were analyzed.
- Subcutaneous density—the brightness of the subcutaneous tissue is expected to increase after the procedure, ie, the opposite impact that is seen in the dermis, and is indicative of a faster recovery. Importantly, it was possible to identify subcutaneous fibrous banding represented by brightness related to density

**Figure 3.**
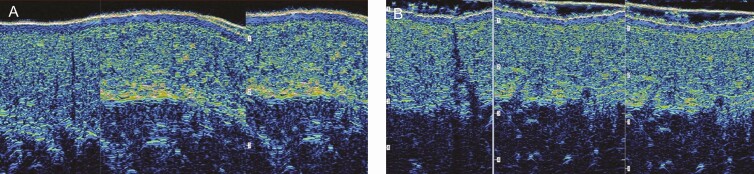
Ultrasound images of an abdominoplasty 3 to 6 weeks postoperative, demonstrating decreased fibrous banding on the R&R side. (A) bland moisturizer and (B) R&R (ReFORM & RePAIR with TriHex Technology [Alastin Skincare, Inc., Carlsbad, CA]).

Seven participants were analyzed at clinical site 1 that had the following procedures: abdominoplasty, vaser liposuction of the hips and flanks, bilateral breast reduction, and mechanical liposuction of the axilla. Five of the measured procedural areas were able to be analyzed and compared; images that had artifacts were excluded from the analysis. All cumulative differential trends were calculated between the 2 sides. In all 5 cases (as with the clinical interpretation), the differential changes appeared from POD 10-14 onwards with clear differences at weeks 3-6. Figure 3 shows ultrasound images of an abdominoplasty postoperative images at 3 to 6 weeks demonstrating decreased fibrous banding on the R&R side (Figure 3A, R&R; Figure 3B, bland moisturizer). At clinical site 2, four participants underwent lateral thigh liposuction and were analyzed. In 3 of the 4 cases, differences appeared from 2 weeks onwards with the bland moisturizer recovering at a slower rate than the R&R side.

### Biopsies

**Figure 4.**
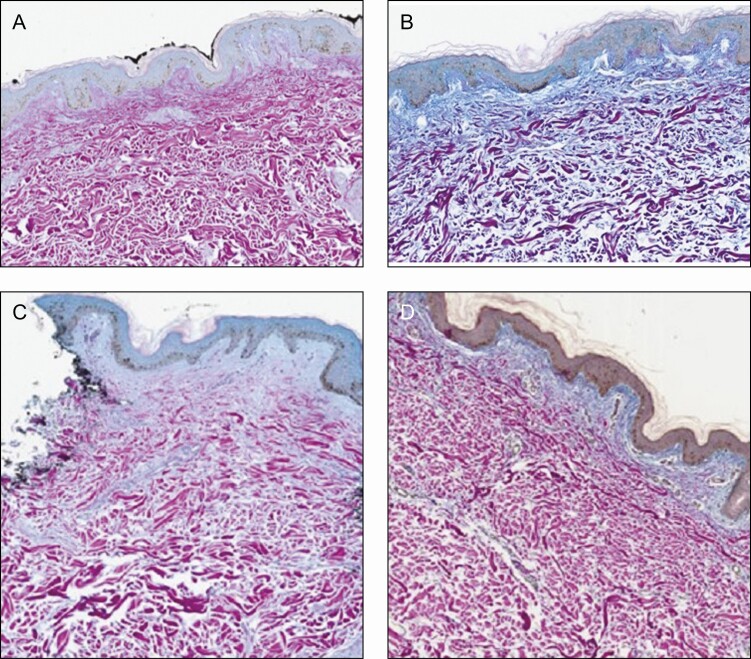
Neocollagenesis with improved extracellular matrix changes was shown as superior to the comparator in all 3 biopsies on the R&R side. R&R: (A) preoperative and (B) 2 weeks postoperative; bland moisturizer: (C) preoperative and (D) 2 weeks postoperative. R&R, ReFORM & RePAIR with TriHex Technology (Alastin Skincare, Inc., Carlsbad, CA).

Five participants consented to biopsies pre topical application and at surgery, of which 3 were of sufficient quality to compare both sides. A blinded independent dermatopathologist (Laboratory and Pathology Diagnostics Naperville, IL) evaluated the biopsies for differences in the skin. The purpose was to document changes associated with preconditioning of the skin before surgery. As described in previous publications, Herovici stain was used to demonstrate a new mucopolysaccharide representation of neocollagenesis.^8^ This is represented by a color change from magenta to blue as demonstrated in Figure 4. Neocollagenesis with improved extracellular matrix changes was shown as superior to the comparator in all 3 biopsies on the R&R side.

### Breaking the Blind

As instructed by the IRB, participants were permitted to break coded blinded topical, if requested. Seven participants broke the blind and requested to apply R&R to both sides. There were no participants who requested to break the blind on the bland moisturizer side. Five participants having the following procedures: abdominoplasty with vaser liposuction to the hips and flanks, bilateral breast reduction with mechanical liposuction to the axilla, and bilateral mastopexy with mechanical liposuction to the axilla and upper abdomen requested to break the blind between POD 10-14 and POD 21-25. One requested during POD 1-3, laser liposuction of the neck, and one at POD 42-50, primary breast augmentation. On average, depending on the surgical procedure, once the participant started to apply R&R to both sides, it took the other side 4-10 weeks to reach the same assessment and, in some cases, post the 12-week follow-up period.

### Photography

**Figure 5.**
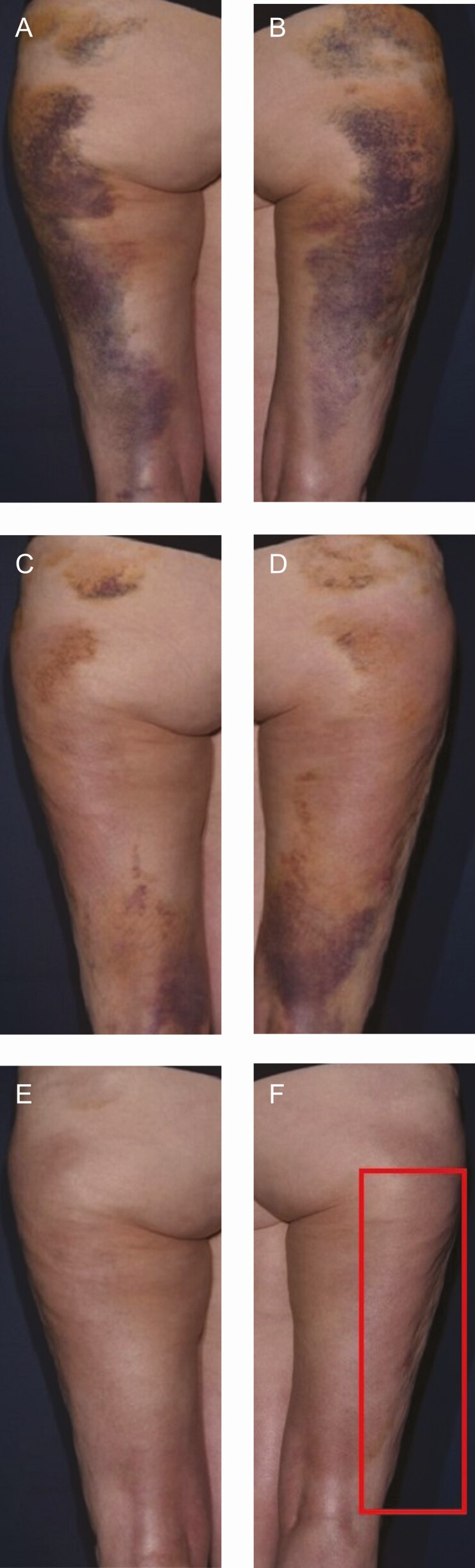

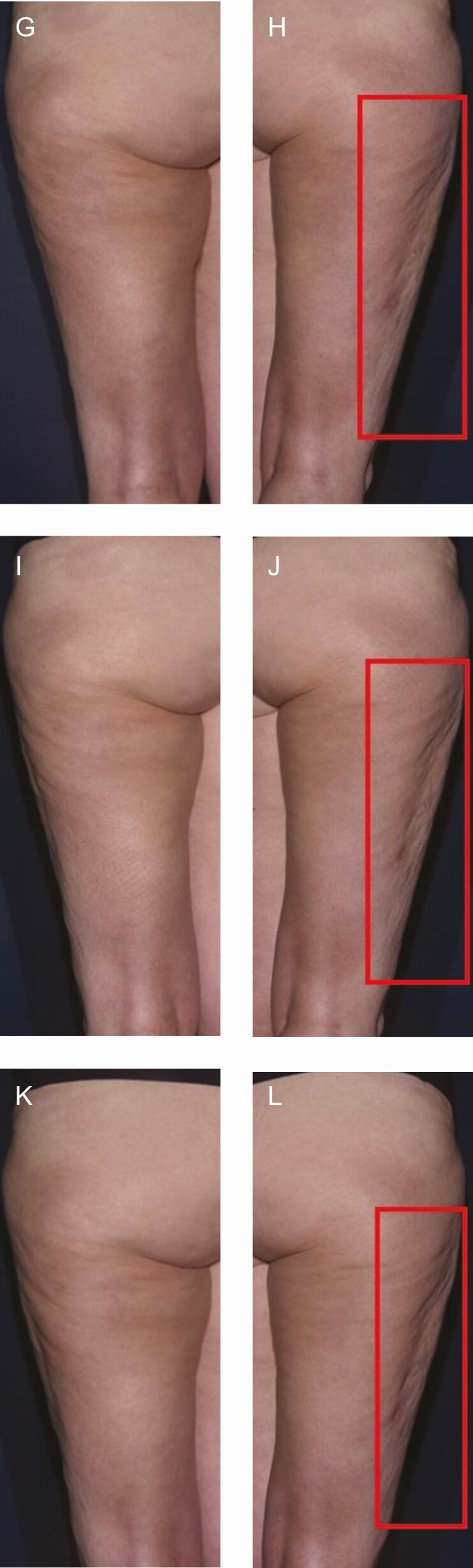
Power-assisted liposuction to the lateral thighs with 250 mL removed bilaterally in a 69-year-old female patient. (A) POD 5-7 on the left side using R&R; (B) POD 5-7 on the right side using bland moisturizer; (C) POD 10-14 left side using R&R, improvement in ecchymosis can be seen; (D) POD 10-14 on the right side using bland moisturizer; (E) POD 21-25 on the left side using R&R, decreased skin discoloration and fibrous banding can be seen; (F) POD 21-25 on the right side using bland moisturizer; (G) POD 28-30 on the left side using R&R, continued decrease in skin discoloration and fibrous banding can be seen; (H) POD 28-30 on the right side using bland moisturizer, fibrous banding continuing through Week 12; (I) POD 42-50 on the left side using R&R; (J) POD 42-50 on the right side using bland moisturizer, fibrous banding continuing through Week 12; (K) Week 12 on the left side using R&R; (L) Week 12 on the right side using bland moisturizer, continuous fibrous banding can be seen. POD, postoperative days; R&R, ReFORM & RePAIR with TriHex Technology (Alastin Skincare, Inc., Carlsbad, CA).

**Figure 6.**
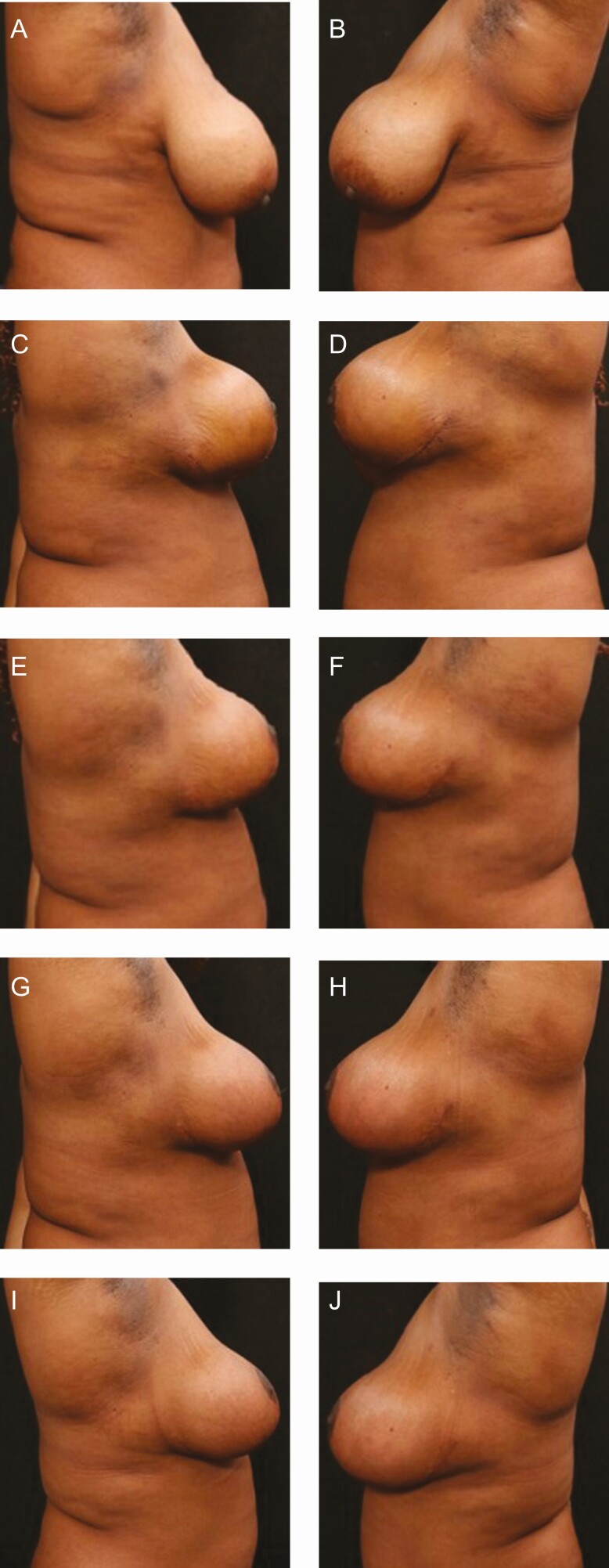
Bilateral mastopexy with mechanical liposuction axilla and upper abdomen with 400 mL removed bilaterally in a 39-year-old female patient. (A) Preoperative on the right side using bland moisturizer; (B) preoperative on the left side using R&R; (C) POD 12 on the right side using bland moisturizer; (D) POD 12 on the left side using R&R, less edema and post traumatic hyperpigmentation in the left axilla can be seen. The authors broke the blind to use R&R on both sides. (E) POD 24 on the right side using bland moisturizer, continued skin discoloration and fibrous banding can be seen; (F) POD 24 on the left side using R&R; (G) Postoperative at 6 weeks on the right side using bland moisturizer; (H) postoperative at 6 weeks on the left side using R&R; (I) postoperative at 12 weeks on the right side using bland moisturizer; (J) postoperative at 12 weeks on the left side using R&R. POD, postoperative days; R&R, ReFORM & RePAIR with TriHex Technology (Alastin Skincare, Inc., Carlsbad, CA).

**Figure 7.**
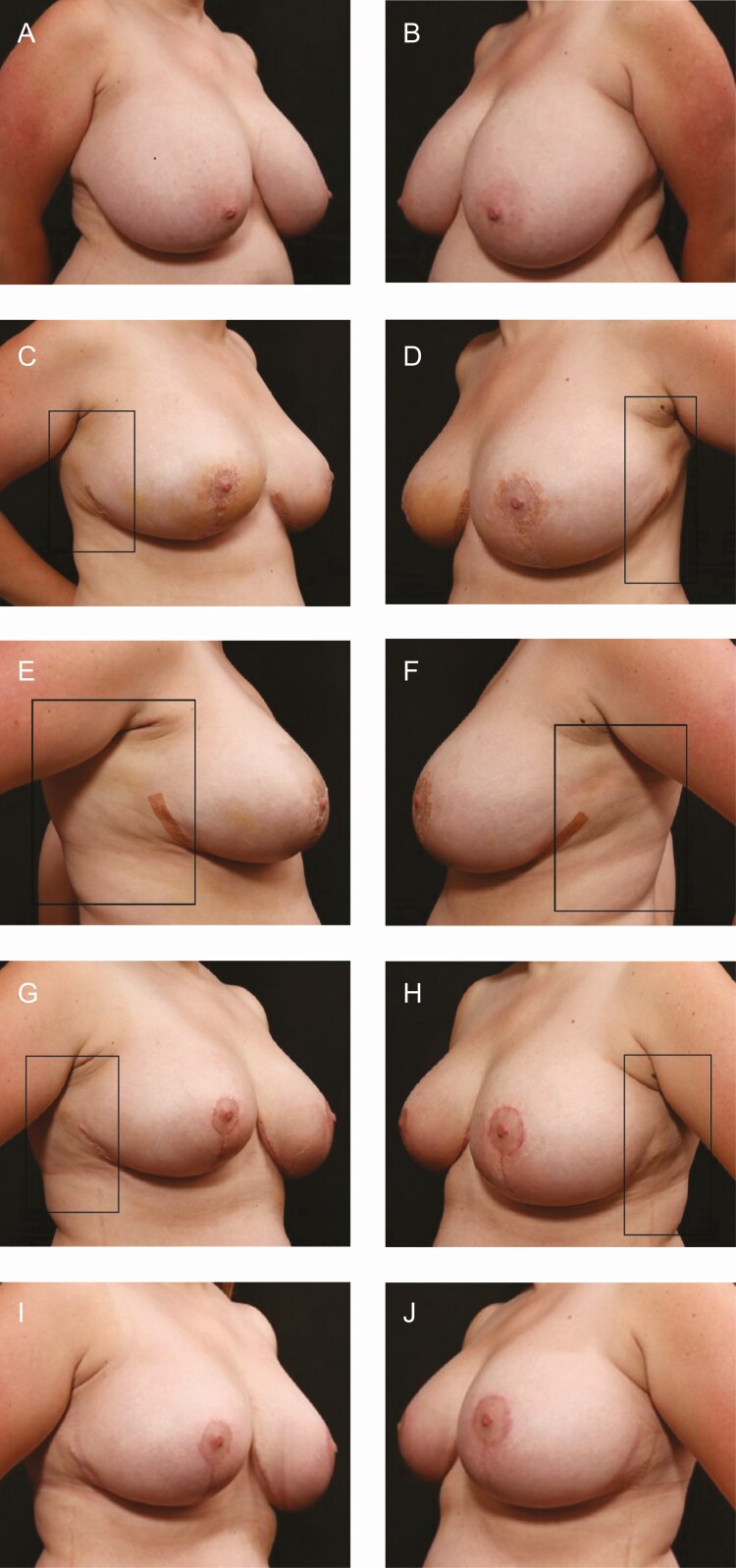
Bilateral breast reduction and liposuction axilla and upper abdomen, and liposuction without heat aspirate 800 mL in a 36-year-old female patient. (A) Preoperative on the right side using R&R; (B) preoperative on the left side using bland moisturizer; (C) postoperative at 2 weeks on the right side using R&R, improved fibrous banding and induration can be seen. The authors broke the blind to use R&R on both sides; (D) postoperative at 2 weeks on the left side using bland moisturizer; (E) postoperative at three-and-a-half weeks on the right side using R&R; (F) postoperative at three-and-a-half weeks on the left side using bland moisturizer, more skin discoloration, edema, and induration can be seen; (G) postoperative at four-and-a-half weeks on the right side using R&R; (H) postoperative at four-and-a-half weeks on the left side using bland moisturizer, more induration and banding can be seen; (I) postoperative at 12 weeks on the right side using R&R; (J) postoperative at 12 weeks on the left side using bland moisturizer. POD, postoperative days; R&R, ReFORM & RePAIR with TriHex Technology (Alastin Skincare, Inc., Carlsbad, CA).

**Figure 8.**
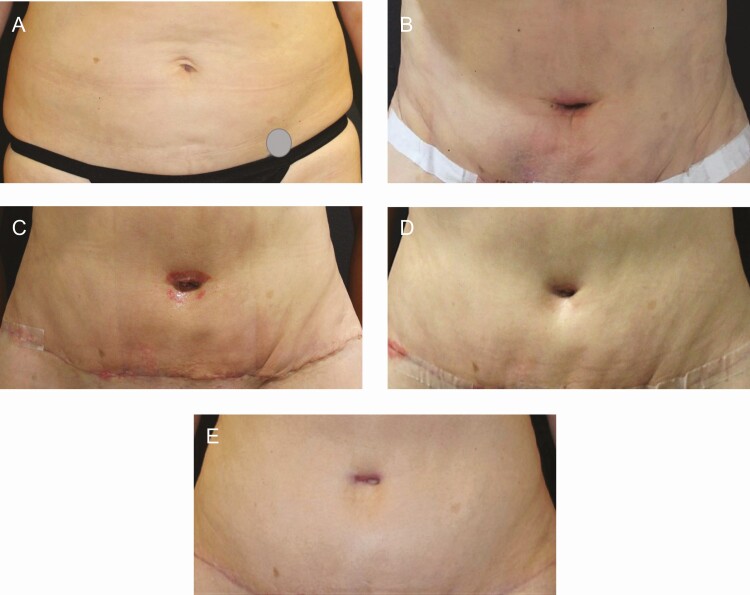
Abdominoplasty with power-assisted liposuction (1200 mL removed) in a 38-year-old female patient. (A) Preoperative; (B) POD 10-14 on the right side using R&R, improvement in the fibrous banding can be seen; (C) POD 28-30 on the right side using R&R, improvement in the fibrous banding can be seen; (D) POD 42-50 on the right side using R&R, improvement in the fibrous banding can be seen; and (E) week 12 with R&R on the right side and bland moisturizer on the left side. POD, postoperative days; R&R, ReFORM & RePAIR with TriHex Technology (Alastin Skincare, Inc., Carlsbad, CA).

Figures 5-8 show participant photography demonstrating improved recovery on the R&R side.

### Safety

There were no associated adverse events with either R&R or the bland moisturizer topical products. Reported adverse events, n = 2, were secondary to the surgical procedure and/or postop medications.

## DISCUSSION

The concept of utilizing a topical formulation to prepare the skin before invasive surgery and immediately afterward is now established with multiple publications demonstrating benefits to recovery and outcomes.^1-7^ The first pilot study compared 2 cohorts of patients undergoing similar noninvasive and invasive body contouring procedures. One cohort used a topical body treatment with tripeptide and hexapeptide, TransFORM Body Treatment with TriHex Technology (TFB; Alastin Skincare, Inc., Carlsbad, CA), and the other cohort did not use a topical. The cohort using TFB had improvement in visible and palpable skin quality and demonstrated postoperatively, reduced PROMs of swelling, induration, soft tissue fibrous banding, and pain compared with the control cohort.^3^ The second split-body clinical study, participants underwent medial thigh liposuction and demonstrated that pre- and post-topical treatment with both RSN and TFB accelerated recovery compared with the control side. Results were confirmed from blinded investigator and participant assessments, ultrasound imaging, induration measurements, and histology of skin biopsies, demonstrating extracellular remodeling and histological evidence of improved collagenesis and elastogenesis.^2^ Furthermore, the results of gene expression studies demonstrated the molecular evidence for the clinical observations of accelerated postprocedure healing in participants using RSN and TFB,^5^ preprocedure and postprocedure compared with the control side. In a third split-body clinical study, participants underwent surgical neck and body contouring procedures and were randomized to apply TFB and RSN to one side and only TFB to the other side. Participants had statistically significant improvements in measures of edema, induration, and subcutaneous fibrous banding on the side that used the combination of topical treatments TFB and RSN 10-14 days preprocedure and 12 weeks postprocedure compared with the side only using TFB.^1^ Other clinical studies have shown that wound bed preparation using topical RSN preprocedure and postprocedure accelerates healing from laser injury and provides evidence of extracellular matrix remodeling when used as preconditioning before aesthetic surgical procedures.^6,7^ The hexapeptide-11 component of TFB has been demonstrated to accelerate (upregulate) the process of autophagy, encouraging lipid droplet breakdown.^4,9-11^ In vitro modeling showed macrophage recruitment to damaged fat cells with clinical trials confirming increased and hastened fat volume reduction.^9,10^ Gene expression studies demonstrated that pre procedural and postprocedural topical treatment with RSN and TFB stimulated extracellular remodeling and induced anti-inflammatory genes, leading to less postprocedural induration supported by ultrasound imaging and analyses.^5^ The third topical product, INhance with TriHex Technology (Alastin Skincare, Inc., Carlsbad, CA), has been validated through in vitro studies to increase the elimination of blood byproducts created from trauma and clinically demonstrated to lessen bruising and edema compared with placebo, postprocedure.^12^

Most of these publications involved the use of 2 separate formulations.^1-3,5^ After the successful introduction of an additional formulation aimed at reducing bruising and swelling used as an adjunct to injection procedures,^12^ a combination product was formulated using the actives from the products in the studies mentioned above. Thus, the active components that had demonstrated efficacy in extracellular matrix remodeling, autophagy, and regenerative macrophage polarization were combined together with components to aid in the appearance of scarring, into one single product.^4^

The process of adipocytolysis is believed to result in the release of inflammatory mediators and stimulation of toll receptors resulting in signs of inflammation including edema, swelling, skin induration, and subsequent fibrous banding.^4^ It is this fibrous banding that results in maximal patient discomfort and restriction of movement weeks following body contouring surgery. Based on these observations, this multicenter trial was undertaken to determine if these longer-term side effects could be limited and to determine if the efficacy of one combined product was comparable to the positive outcomes previously reported with a combination of different products.

As with the previous studies, in which gene expression and side-effect profiles started to show differences at the 2-week follow-up, this trial demonstrated the first significant differences in ecchymosis at this time point. Gene expression studies also revealed that extracellular matrix (ECM) remodeling and healing profiles were markedly improved compared with the bland topical at 4 weeks. In that study, the profiles were examined at 2 and 4 weeks. In this larger multi-site trial with more time point measurements, the most important sign of internal scarring, that of fibrous banding, showed significant differences at 3, 4, 6, and 12 weeks. In addition, induration and edema also showed significant differences after the 2-week time point. The initial improvement in ecchymosis at 2 weeks was followed by improvement in skin discoloration in subsequent weeks, a reflection of the blood products and hemosiderin being absorbed, and the normalization of color from purple to reddish-brown to resolved over time.

Participants also confirmed the findings of improvement in skin discoloration and fibrous banding over very similar time periods. In addition, 7 patients broke code, all on the R&R side which is probably the strongest reflection of subject preference and efficacy of the product. Thus, overall, a convincing picture of improved efficacy was demonstrated between the test product and bland moisturizer which manifested in hastened healing, decreased discomfort, and reversal of movement limitation. This translated to a healing advantage of 4 to 8 weeks with varying results from different procedures. The more invasive the procedure, the more use of bovie, electrosurgical, rather than cold steel and the more disruption of fatty compartments, the more significant the improvement in healing. This was also reflected in the ultrasound results, where recovery advantages were noted from approximately the 2-week follow-up period, as opposed to abdominoplasty and breast reduction, which started showing definite improvement over the comparator from the 3-week follow-up. To assess whether the patient had limitations in range of motion from subcutaneous banding, the patient was asked to raise their arms overhead and fully stretch to the right and to the left. On the full stretch, the patient was asked to palpate the subcutaneous tissue on the contralateral side of the stretch to evaluate the subcutaneous banding. Patients often could not stretch completely due to discomfort from the pulling feeling and discomfort to the overlying skin. At clinical site 1, the patients all had MLD followed by deep tissue release at each postop visit to both operated sides. The blinded assessor noted that patients required 50% less massage time on the side treated with R&R than with the bland topical. An additional clinical site reported 25% less MLD.

As detailed in previous publications, surgical procedures, especially those involving body contouring surgery, elicit adipocytolysis, characterized by inflammation arising from the disruption of adipocytes through the direct action of proinflammatory cytokines or catecholamines.^9^ Cell death by necrosis results in the release of saturated fatty acids which act as endogenous molecules known as damage-associated molecular patterns, capable of activating toll-like receptor-induced macrophage activation, leading to the production and liberation of proinflammatory cytokines.^9^ It is surmised that the increased effective mobilization, autophagic repackaging, and macrophage absorption of these droplets manifests in the hastened healing, with less induration, and resolution of internal scarring, manifesting as subcutaneous fibrosis banding. These postoperative changes appear to be accentuated by preoperative preparation of the skin improving cellular signaling and ECM remodeling. This has been documented in previous studies^2,6,7^ and to a lesser degree was confirmed with the few biopsies completed in this study as an additional component.

Although this study was a split-body evaluation, limitations included differentiation between surgical garments and dressings at the clinical sites and MLD postoperatively, depending on the clinical site and the postop care. The clinical sites that performed MLD reported less time needed on the R&R side.

## CONCLUSIONS

A multicenter trial was undertaken to test the efficacy of a product used before and after body contouring surgery formulated with a combination of active ingredients previously validated in in vitro and in vivo tests for various indications. The common goal was the elimination of “waste products” in the form of lipid droplets, blood products, and damaged cellular components, which allowed hastened healing and more rapid resolution of surgical signs and symptoms. The trial demonstrated a significant difference in ecchymosis, skin discoloration, edema, and importantly, ongoing resolution of induration and fibrous banding over many weeks. The use of a topical product to aid in invasive surgical healing resolution is a new concept; although other researchers have used the preconditioning concept pertaining to scar control,^13^ this clinical validation provides exciting new avenues for exploration and an additional adjunct to surgical procedures.
